# Clinical prognosis of intraoperative blood salvage autotransfusion in liver transplantation for hepatocellular carcinoma: A systematic review and meta-analysis

**DOI:** 10.3389/fonc.2022.985281

**Published:** 2022-10-18

**Authors:** Zheng Wang, Saixin Li, Yitong Jia, Miao Liu, Kun Yang, Minghao Sui, Dongbin Liu, Kuo Liang

**Affiliations:** ^1^ Department of General Surgery, Xuanwu Hospital, Capital Medical University, Beijing, China; ^2^ Department of Anesthesiology, Xuanwu Hospital, Capital Medical University, Beijing, China; ^3^ Department of Evidence-Based Medicine, Xuanwu Hospital, Capital Medical University, Beijing, China

**Keywords:** hepatocellular carcinoma, intraoperative blood salvage autotransfusion, liver transplantation, leukocyte depletion filters, treatment outcome, meta-analysis.

## Abstract

**Background:**

Intraoperative blood salvage autotransfusion(IBSA) has been widely used in a variety of surgeries, but the use of IBSA in hepatocellular carcinoma (HCC) patients undergoing liver transplantation (LT) is controversial. Numerous studies have reported that IBSA used during LT for HCC is not associated with adverse oncologic outcomes. This systematic review and meta-analysis aims to estimate the clinical prognosis of IBSA for patients with H+CC undergoing LT.

**Methods:**

MEDLINE, Embase, Web of Science, and Cochrane Library were searched for articles describing IBSA in HCC patients undergoing LT from the date of inception until May 1, 2022, and a meta-analysis was performed. Study heterogeneity was assessed by I^2^ test. Publication bias was evaluated by funnel plots, Egger’s and Begg’s test.

**Results:**

12 studies enrolling a total of 2253 cases (1374 IBSA and 879 non-IBSA cases) are included in this meta-analysis. The recurrence rate(RR) at 5-year(OR=0.75; 95%CI, 0.59-0.95; P=0.02) and 7-year(OR=0.65; 95%CI, 0.55-0.97; P=0.03) in the IBSA group is slightly lower than non-IBSA group. There are no significant differences in the 1-year RR(OR=0.77; 95% CI, 0.56-1.06; P=0.10), 3-years RR (OR=0.79; 95% CI, 0.62-1.01; P=0.06),1-year overall survival outcome(OS) (OR=0.90; 95% CI, 0.63-1.28; P=0.57), 3-year OS(OR=1.16; 95% CI, 0.83-1.62; P=0.38), 5-year OS(OR=1.04; 95% CI, 0.76-1.40; P=0.82),1-year disease-free survival rate(DFS) (OR=0.80; 95%CI, 0.49-1.30; P=0.36), 3-year DFS(OR=0.99; 95%CI, 0.64-1.55; P=0.98), and 5-year DFS(OR=0.88; 95%CI, 0.60-1.28; P=0.50). Subgroup analysis shows a difference in the use of leukocyte depletion filters group of 5-year RR(OR=0.73; 95%CI, 0.55-0.96; P=0.03). No significant differences are found in other subgroups.

**Conclusions:**

IBSA provides comparable survival outcomes relative to allogeneic blood transfusion and does not increase the tumor recurrence for HCC patients after LT.

**Systematic review registration:**

https://www.crd.york.ac.uk/prospero/, identifier CRD42022295479.

## Introduction

Hepatocellular carcinoma (HCC) is the most frequent primary liver cancers, the sixth most common neoplasm, and the third most common cause of cancer death (1). Liver transplantation(LT) is the most curative treatment for HCC on cirrhosis in the absence of metastases and macroscopic vascular invasion, as it effectively treats both the tumor burden and the underlying liver disease. Milan criteria established LT as a valid treatment option for HCC patients with cirrhosis (2, 3). However, elevated portal pressure, increased collateral circulation and the hyperdynamic, dilated, thin-walled splanchnic circulation all contribute to an increased risk of hemorrhage during the LT which are distinct causes of bleeding that are different from those in other surgeries (4). Intraoperative hemorrhage has been recognized as a mortality risk, necessitating massive blood transfusions during LT (5).

Blood transfusion could be divided into autotransfusion and allogenic blood transfusion (ABT) based on the blood source. Three types of autologous transfusion exist: prestored autotransfusion, dilution autotransfusion, and intraoperative blood salvage autotransfusion(IBSA). ABT is the primary technique employed in conventional application, but it may transmit hepatitis virus and human immunodeficiency virus, as well as cause an immunological transfusion reaction (6, 7). Noninfectious risks are also well known, such as transfusion-associated circulatory overload and acute lung injury. In particular, ABT may impair the immune function of tumor patients (8), which could increase the risk of postoperative infections, lengthen hospital stays, and, in severe circumstances, even result in death (9). With the rising demand for clinical blood, the shortage of blood supply and the underlying risk of transfusion of banked blood, autologous blood transfusion is becoming more common in clinics to avoid or reduce the risks associated with ABT (10, 11).

The use of IBSA in HCC patients involving LT is controversial, the critical point is whether IBSA increases the risk of recurrence or metastasis due to reperfusion of tumor cells (12, 13). Even though this hypothesis is unwarranted, it still limits the utilization of IBSA. Foltys et al. have demonstrated IBSA does not modify the risk of HCC recurrence, the use of IBSA appears to be justified in highly selected HCC patients undergoing LT (14), and the European Society of Anesthesiology does not contraindicate its use in cancer patients (15), but there is still no consensus on its usage in patients undergoing LT for HCC (16). Since the published results were largely based on a retrospective analysis of cases from a single center, and randomized controlled trials (RCTs) are difficult to conduct in this setting, we conduct this meta-analysis to fully estimate the clinical prognosis of IBSA for patients with HCC undergoing LT which may be helpful in elucidating the issue.

## Methods

This systematic review and meta-analysis adhere to the Preferred Reporting Items for Systematic Reviews and Meta-Analyses(**Supplementary Table 1**) and has been registered with the International Prospective Register of Systematic Review(PROSPERO) database (registration number CRD42022295479) (17). This systematic review is conducted using the methodological guidance in the Cochrane Handbook for Systematic Reviews of Interventions (18). Any modifications to this protocol made over the course of the study will be reported in PROSPERO and the final manuscript.

### Study identification and selection

The search strategies were created by an investigator (KY) with database search experience. We conducted database searches in the following databases: Medline (via PubMed), Web of Science databases, EMBASE and The Cochrane Library. Databases were used to identify suitable studies that were published up to 1 May 2022. Three search themes were combined with the Boolean operator ‘and’ in searching databases, and the search terms were as follows: ‘Autotransfusion’, ‘Liver Transplantation’ and ‘Hepatocellular Carcinoma’. Detailed search strategy was shown in **Supplementary Methods**. Only English-language publications with human subjects were included in the searches. The following inclusion criteria were used: (a) a study that investigated the clinical prognosis during LT for HCC patients; (b) randomized clinical trial, high-quality case–control study, cohort study; (c) adults (over the age of 18). The exclusion criteria were as follows: (a) comments, case reports, and letters to the editors; (b) duplicate reports; (c) systematic reviews or meta-analyses. Two reviewers(YJ and SL)independently screened the articles according to the inclusion criteria. In case of discrepancies, consistencies will be ensured by a third reviewer(ZW). If several studies present data from the same study population, or multiple publications from the same research series are published in chronological sequence, the study with the most direct interventions or the largest sample size was kept.

### Data extraction and quality assessment

The following parameters were extracted from the full-text article: the name of the first author, periodical titles, country, publication year, type of study, characteristics of IBSA group and non-IBSA group (eg, age, sex, follow-up years, sample size, overall survival outcome, disease-free survival outcome, recurrence rate and any adverse events caused by the preventive interventions). Two reviewers(YJ and DL) extracted data from studies in accordance with the screening process, and any inconsistencies were resolved by a third reviewer(SL). In case of any ambiguity or insufficient information, wherever possible, authors of primary studies were contacted by either telephone, email or post to obtain missing data. We made a summary sheet containing all the data fore-mentioned. On the other hand, we assessed the quality of published literature by two independent reviewers (ZW and YJ). The risk of bias of RCTs was assessed with items in the Cochrane Collaboration’s tool (19). Non-RCTs (observational cohort and case-control studies) were assessed with the Newcastle-Ottawa Scale (20). Studies were classified as poor quality if their quality scores fell below 7, which was the threshold for high quality studies.

### Outcome measures

The primary outcome of this meta-analysis is the tumor-related recurrence rate of use IBSA during LT for HCC. The recurrence time points will be 1-year, 3-year, 5-year and 7-year after LT. Radiological data was used to determine whether HCC had recurred (21). Other survival outcomes, such as the overall survival and the disease-free survival, if available, would also be analyzed and reported.

### Statistical analysis

Meta-analyses were conducted when appropriate using Review Manager 5.4 and STATA 16.0 statistical software. For each outcome, odds ratio (OR) and corresponding 95% confidence intervals(CI) were used to measure the association for each study. We will apply mathematical operations to convert data that is presented in the literature as median and quartiles into mean and standard deviation format (22). Forest plots will be used to visualize pooled estimates and the extent of heterogeneity among studies. The I^2^ statistic were used to assess statistical heterogeneity among the included studies (I^2^ values of <40%, 40%–60%, 50%-90%, and 75%-100% represent mild, moderate, substantial and considerable heterogeneity, respectively) (23). I^2^ > 50% will be considered as having a substantial heterogeneity, the random-effects model (the DerSimonian and Laird method) will be used to analyze the outcomes, otherwise, a fixed-effect model(the Mantei-Haenszle method) would be applied. The sources of heterogeneity will be explored by using sensitivity analyses. A subgroup analysis will be conducted to determine whether the results differed according to the use of leukocyte depletion filters (LDFs). The potential for publication bias will be assessed by the funnel plot, Egger test and Begg’s test (24–26).

## Results

**Figure 1:**
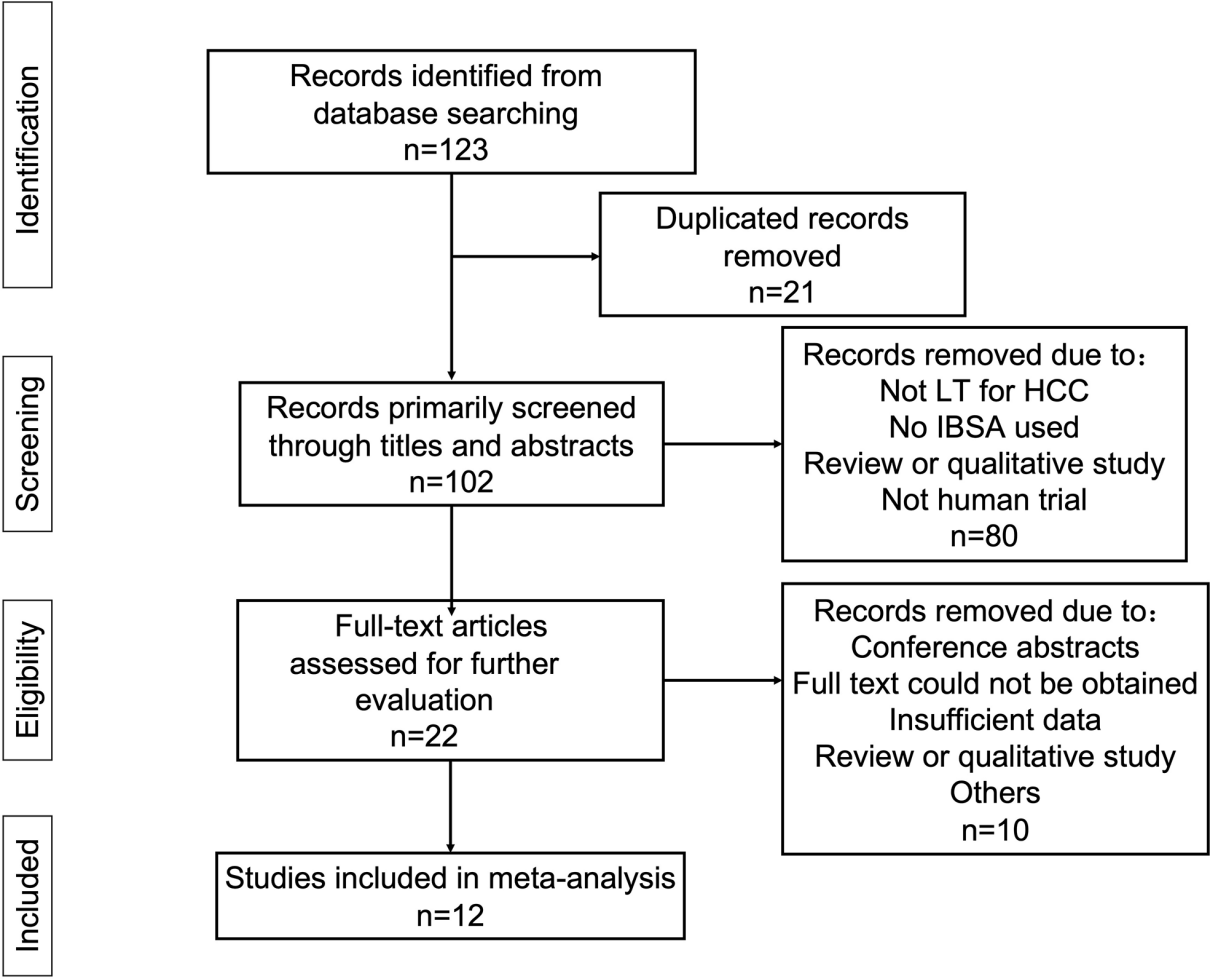
Flow diagram of the selection and screening process for eligible studies.

The database searches returned 123 results, 22 of which were excluded due to duplication. Further, 34 studies were excluded because they were reviews or qualitative study or were not relevant to the topic being studied. The remaining articles were fully read. Finally, 12 studies enrolling a total of 2253 cases (1374 IBSA cases and 879 non-IBSA cases) were included in the meta-analysis (14, 27–37). The process used for article selection is presented in **Figure 1**.

The selected studies had been published between 2005 and 2022. The sample size of studies ranged from 23 to 397. All of the studies were cohort studies. There were no randomized controlled trials. According to the Newcastle-Ottawa Scale, most (n = 11, 91.67%) of the studies were defined as high-quality studies (score more than 7), the detailed assessments are shown in **Supplement Table 2**. The baseline characteristics of the included studies are presented in **Table 1**.

**Table 1:** Characteristics of included studies.

Study	Year	Study type	LDFs used	>Sample size			Age ( year, Mean ±SD)		Sex, male		Outcome	NOS score
				IBSA group	Non-IBSA group		IBSA group	Non-IBSA group	IBSA group	Non-IBSA group		
Akbulut (27)	2013	RCS	0	24		59	52.0±1.8	51.0±1.2	22	52	RR,OS,DFS	7
Araujo (28)	2016	RCS	1	122		36	57.9±2.1*	61.8±1.4*	95	27	RR,OS	8
Foltys (14)	2011	RCS	1	40		96	54.9±6.6*	59.8±7.6*	28	74	RR,OS	7
Han (29)	2016	RCS	1	283		114	31.9±11.2	30.7±1.3	197	77	RR	9
Ivanics (30)	2021	RCS	0	76		34	56.0±1.9*	54.7±2.6*	61	30	RR,OS	8
Kim (31)	2012	RCS	1	121		109	52.3±7.1	52.6±7.5	97	86	RR	8
Kwon (32)	2021	RCS	1	220		129	54.0±1.6*	53.0±1.7*	192	121	RR,OS	8
Muscari (33)	2005	RCS	1	31		16	53.0±12.0	58.0±6.0	26	14	RR	7
Nutu (34)	2021	RCS	0	192		186	59.2±7.3	58.4±7.7	NA	NA	RR,OS,DFS	8
Pinto (35)	2021	RCS	0	122		34	59.0±7.0	60.0±6.0	75	20	RR,OS,DFS	7
Sutton (36)	2021	RCS	0	131		55	59.0±1.4*	61.8±1.3*	98	45	RR,OS	7
Weller (37)	2021	RCS	0	12		11	54.8±6.7	58.3±7.4	9	9	RR	6

0, don't use LDFs; 1, use LDFs; DFS, Disease-free survival; IBSA, intraoperative blood salvage autotransfusion; NOS, Newcastle-Ottawa Scale; OS, Overall survival; RR, recurrence rate; RCS, retrospective cohort study; SD, standard deviations.

^∗^Switched to mean ± SD according to the formula of Cochrane handbook.

### Primary outcomes: Tumor recurrence

**Figure 2:**
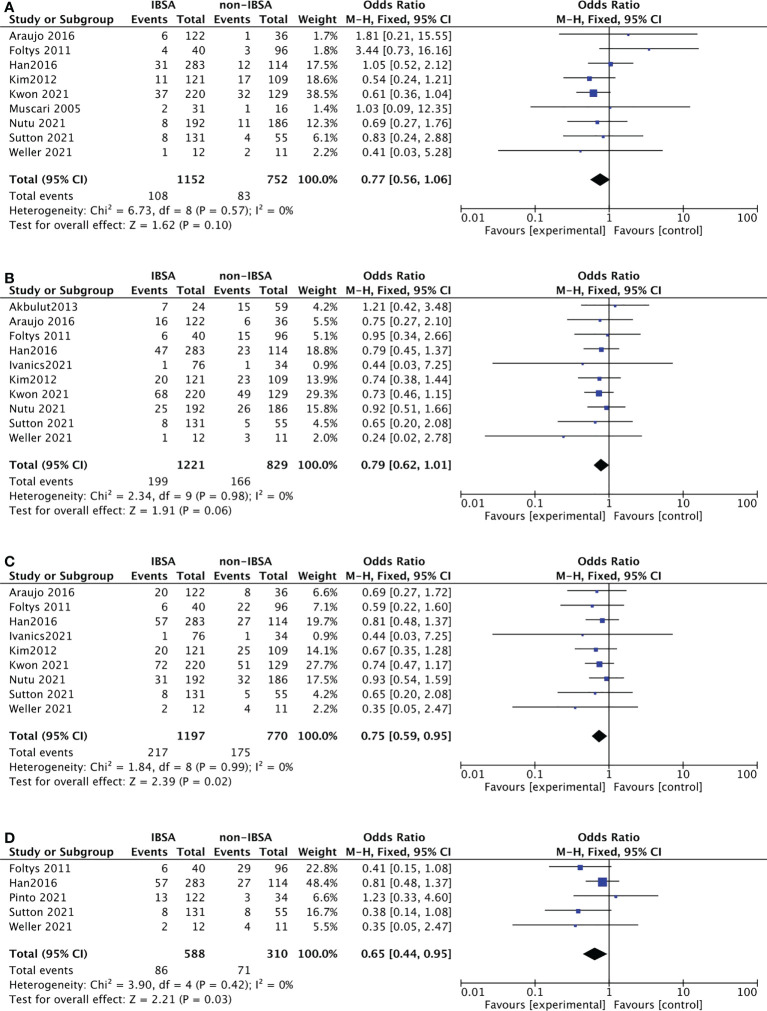
Meta-analysis forest plot of the recurrence rate. **(A)**, 1-year RR; **(B)**, 3-year RR; **(C)**, 5-year RR; **(D)**, 7-year RR.

Twelve studies reported the recurrence rate(RR) outcomes of IBSA and non-IBSA patients. Of them, seven studies provided a specified description of criteria for determining the recurrence and follow-up methods (14, 27, 29, 32, 35–37). The meta-analysis data is displayed in **Figure 2**, the RR at 5-year(OR=0.75; 95%CI, 0.59-0.95; P=0.02) and 7-year(OR=0.65; 95%CI, 0.44-0.95; P=0.03) in the IBSA group was slightly lower than non-IBSA group. There were no significant differences in the 1-, and 3-years RR. The RR at 1-, and 3-year had ORs of 0.77 (95% CI, 0.56-1.06; P=0.10), and 0.79 (95% CI, 0.62-1.01; P=.06), respectively. No heterogeneity was found in 1-year RR (I^2^ = 0%), 3-year RR (I^2^ = 0%), 5-year RR (I^2^ = 0%), and 7-year RR(I^2 =^ 0%), the fixed effect model was adopted.

### Overall survival and disease-free survival

**Figure 3:**
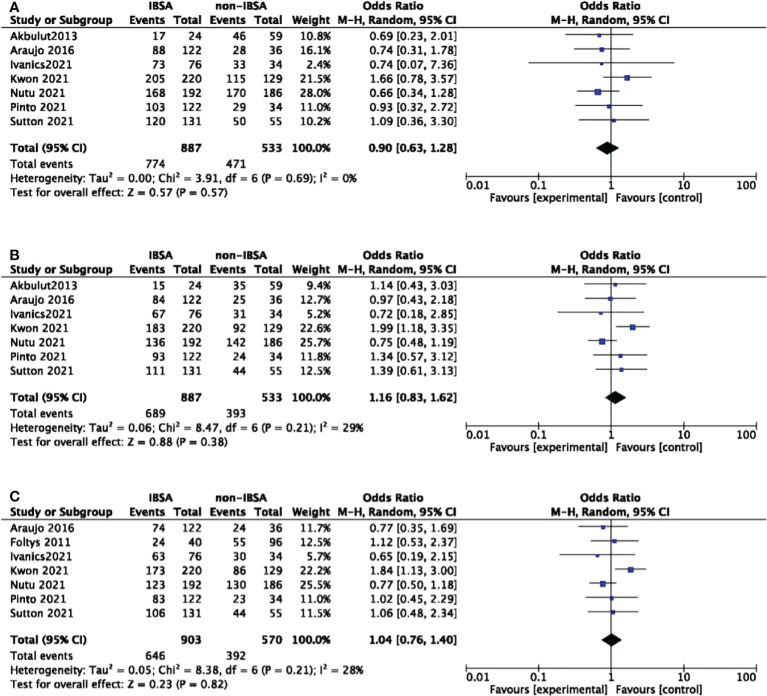
Meta-analysis forest plot of the overall survival. **(A)**, 1-year OS; **(B)**, 3-year OS; **(C)**, 5-year OS.

**Figure 4:**
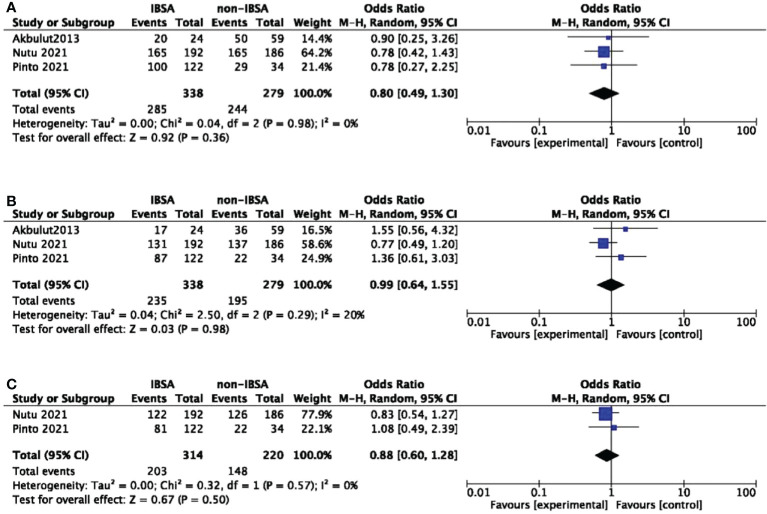
Meta-analysis forest plot of the disease-free survival. **(A)**, 1-year DFS; **(B)**, 3-year DFS; **(C)**, 5-year DFS.

Eight studies reported the overall survival(OS) outcomes of IBSA and non-IBSA patients (**Figure 3**). The overall survival outcomes at 1-, 3-, and 5-year were not significantly different. The OS at 1, 3, and 5-year had RRs of 0.90 (95% CI, 0.63-1.28; P=0.57), 1.16 (95% CI, 0.83-1.62; P=0.38), and 1.04 (95% CI, 0.76-1.40; P=0.82). Mild heterogeneity was observed in 1-year OS (I^2^ = 0%), 3-year OS (I^2^ = 29%), 5-year OS (I^2^ = 28%). For all included studies performed in statistics of disease-free survival(DFS), there were no significant differences at 1-year DFS(OR=0.80; 95%CI, 0.49-1.30; P=0.36), 3-year DFS(OR=0.99; 95%CI, 0.64-1.55; P=0.98), 5-year DFS(OR=0.88; 95%CI, 0.60-1.28; P=0.50) (**Figure 4**). Mild heterogeneity was found in 1-year DFS (I^2^ = 0%), 3-year DFS (I^2^ = 20%), and 5-year DFS (I^2^ = 0%).

### Subgroup analysis

**Figure 5:**
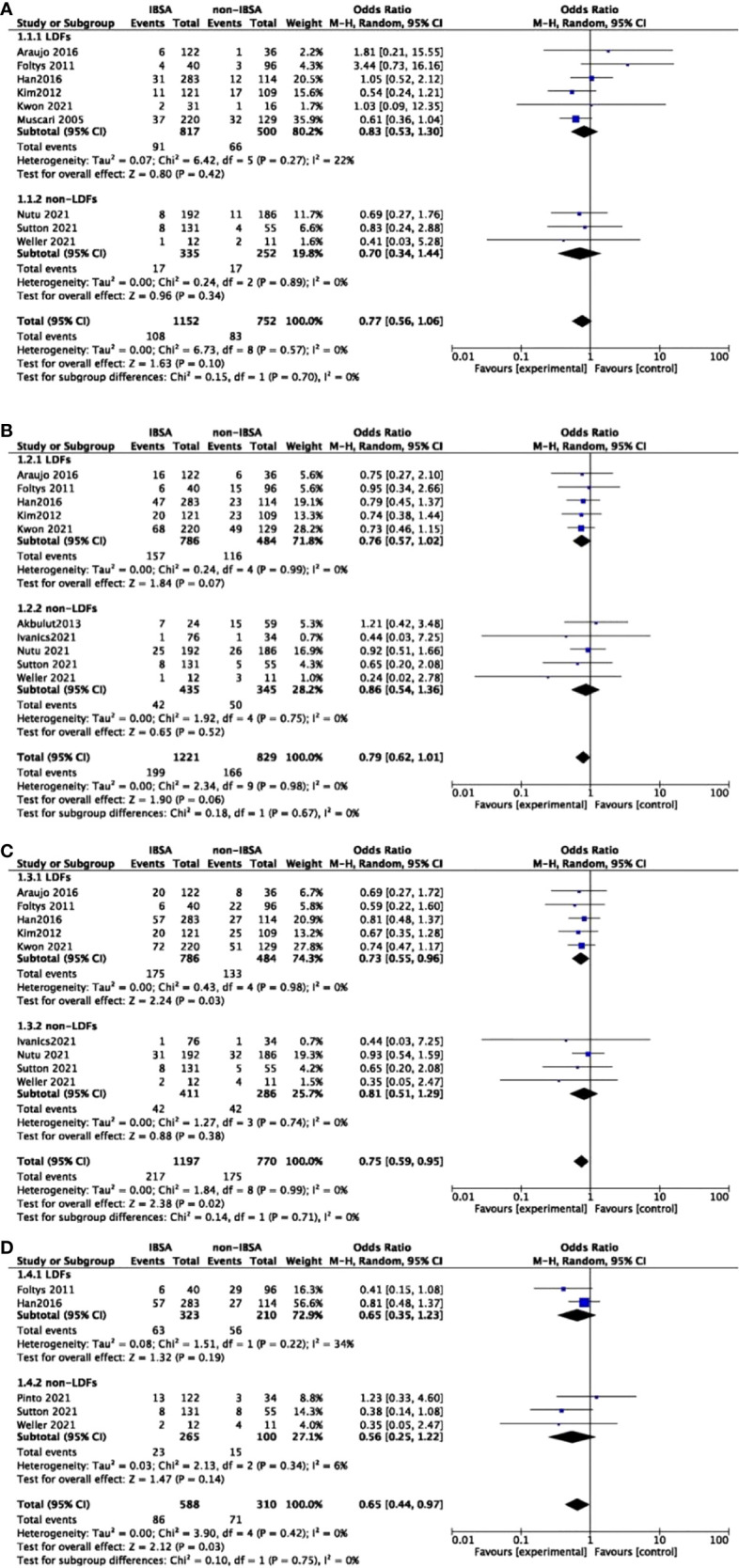
Meta-analysis forest plot of subgroup analysis of the recurrence rate. **(A)**, 1-year RR; **(B)**, 3-year RR; **(C)**, 5-year RR; **(D)**, 7-year RR.

**Figure 6:**
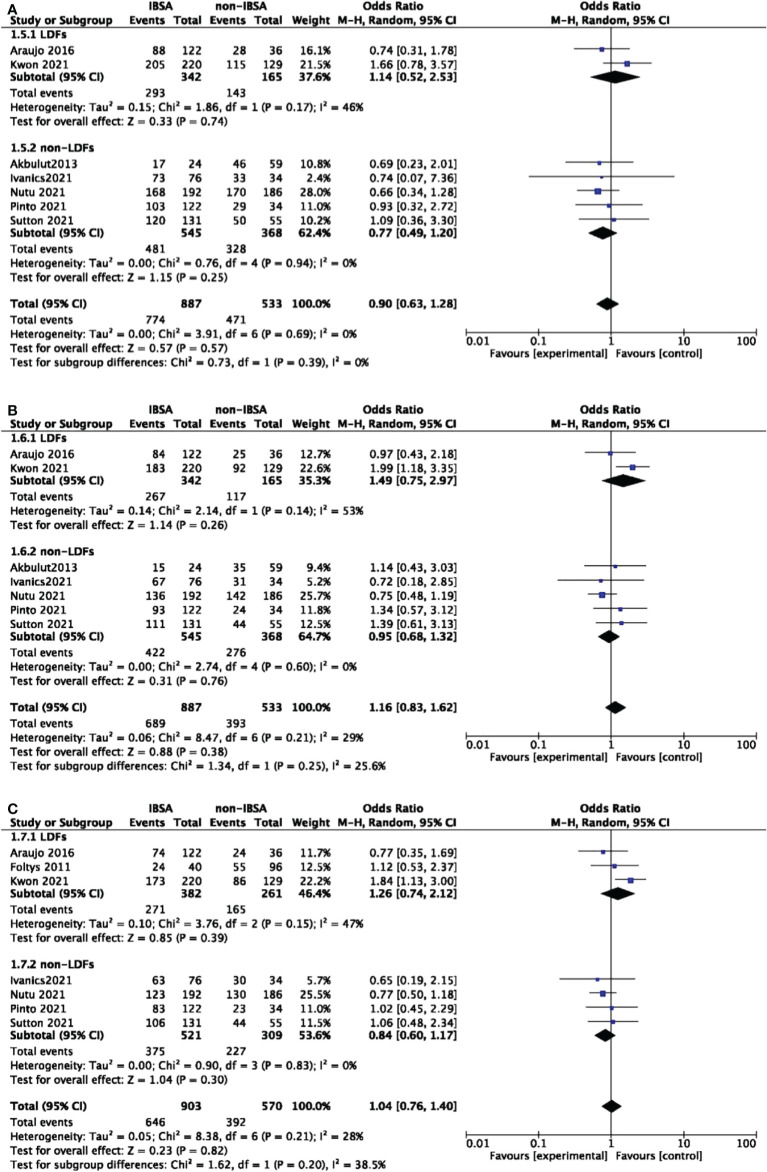
Meta-analysis forest plot of subgroup analysis of the overall survival. **(A)**, 1-year OS; **(B)**, 3-year OS; **(C)**, 5-year OS.

A predesigned subgroup analysis was conducted according to the use of LDFs. Six studies attached LDFs to IBSA during LT (14, 28, 29, 31–33). The RR and OS outcomes were evaluated according to the use of LDFs, DFS was not evaluated due to lack of data. We observed a difference in the LDFs-using group of 5-year RR (OR=0.73; 95%CI, 0.55-0.96; P=0.03). No significant differences were found in other subgroups. Pooled ORs are detailed in **Figures 5**, **6**.

### Sensitivity analysis

For primary outcomes, pooled effects of ORs remained stable after removing any single study at 1-, 3-, 5-, and 7-year RR. For secondary outcomes, the removal of Kwon’s study led to a reduction in heterogeneity at 3-year and 5-year OS (32). Filled pooled effects were adjusted for 3-year OS(OR=0.95; 95%CI, 0.70-1.29; P=0.73), 5-year OS(OR=0.86; 95%CI, 0.65-1.14; P=0.30), which were consistent with the initial meta-analysis. For 1-year OS, OR did not change much by removing either study (**Supplementary Figure 1**). Sensitivity analysis was not performed for DFS due to fewer studies.

### Publication bias

We used Egger’s test and Begg’s test to evaluate the publication bias for RR and OS outcomes. No indication of publication bias was observed for 1-year RR (Egger’s test, P = 0.158; Begg’s test, P = 0.7205), 3-year RR (Egger’s test, P = 0.694; Begg’s test, P = 0.4743), 5-year RR (Egger’s test, P = 0.901; Begg’s test, P = 0.0763), and for 1-year OS (Egger’s test, P = 0.943; Begg’s test, P = 0.8065), 3-year OS (Egger’s test, P = 0.943; Begg’s test, P = 0.7639), 5-year OS (Egger’s test, P = 0.517; Begg’s test, P = 0.7639). Funnel plots were visually examined for symmetry for all outcomes reported (**Supplementary Figures 2**, **3**).

## Discussion

In this comprehensive systematic review and meta-analysis, we identified 12 cohort studies investigating the clinical prognosis of IBSA during LT for HCC. The recurrence rate was used as the primary outcome, and the overall survival and disease-free survival were used as the secondary outcomes. The analyses showed that the RR at 5- and 7-year in the IBSA group was slightly lower than non-IBSA group. No significant differences were found between the IBSA and non-IBSA groups in the 1-, and 3-year RR outcomes. For secondary outcomes, the OS outcomes at 1-, 3-, and 5-year and the DFS outcomes at 1-, 3-, and 5-year were not significantly different. Sensitivity analysis was carried out to evaluate whether the result is stable and reliable, adjusted effects did not fluctuate much by omitting each study. Given the above, though no randomized studies were included, results of the meta-analysis could be considered relatively solid and trustworthy based on the current studies.

The use of IBSA reduces the requirement for allogeneic blood during surgery, preventing adverse transfusion reactions without having a negative impact on other clinical outcomes. However, oncological surgery is still regarded as a relative contraindication to IBSA over concern of reinfusing tumor cells and thereby causing tumor dissemination (13, 38, 39). The presence of neoplastic cells in blood samples from an autotransfusion system in 1975 established a link between the usage of IBSA and the occurrence of metastasis, although there is no proof that these cells have the capacity to cause recurrence or metastasis (40). In our study, IBSA did not increase the tumor recurrence rate and had comparable survival outcomes with non-IBSA. Based on existing literature, the European Society of Anesthesiology does not contraindicate the use of IBSA in patients with cancer (15). Furthermore, a rencent study has demonstrated the effectiveness of IBSA in reducing the need for ABT for LT (41). A sizable prospective analysis that was conducted confirmed the cost effectiveness of IBSA. With the use of autologous transfusion over the study period, a cost saving of $188618 United States dollars was achieved (42). In a multicenter research encompassing more than 33000 individuals, the risk of side effects associated with the usage of IBSA was estimated to range from 0% to 0.006% (11). Even though we need more evidence with large-sample size randomized control studies, those studies suggest that we should reduce the use of ABT.

Subgroup analysis was performed to determine whether results were differed due to the use of LDFs. LDFs were added to IBSA in the 1990s to increase the safety of the procedure (43). But it is still debatable whether LDFs completely decrease the risk of tumor cell metastasis. Several reports have demonstrated that LDFs are effective at eliminating tumor cells *in vitro* and vivo studies (39, 44, 45). However, there have been few reports using HCC cells. Unless there were large cell loads, according to Gwak’s experimental results, LDF could filter HCC cells *in vitro* (46). And LDFs incorporated into cell salvage circuits have shown to effectively remove malignant cells when used during LT of patients with non­ruptured hepatocellular tumors (16). Those studies support the hypothesis that tumor cells could be efficiently removed during collection, processing, and leukocyte filtration.

Six studies included in this meta-analysis attached LDFs to IBSA, in the subgroup analysis, IBSA-group has a low 5-year RR than non-IBSA group with the use of LDFs. This might be as a result of ABT’s effect on immune function of patients with tumors. Besides 5-year RR outcome, non-LDFs-using group had similar results as the LDFs group. The above studies are insufficient to explain the adverse effects of the presence of tumor cells on clinical prognosis and to demonstrate negative effects associated with the use of IBSA. Some organizations, including the National Institute of Clinical Excellence, the Association of Anaesthetists of Great Britain and Ireland and the American College of Obstetricians and Gynecologists have developed guidelines to support the use of IBSA or in combination with LDFs in cancer surgery (14, 47–49). The findings in this study imply that using LDFs in combination may be a preferable way.

To our knowledge, a meta-analysis included eleven studies suggests that cancer recurrence after the use of IBSA is not inferior to traditional intraoperative allogeneic transfusion, with an odds ratio of 0.65 (95% CI, 0.43-0.98; P = 0.0391). But the included studies of this meta-analysis ranged from different cancer types, only three studies involved patients with hepatocellular carcinoma (50). In addition, another meta-analysis included 9 studies demonstrated that IBSA did not increase the tumor recurrence rate and had comparable survival outcomes with ABT. In the subgroup analysis of five studies for liver cancer surgery, IBSA did not increase the mortality risk with long-term follow-up for patients with hepatocellular carcinoma (51). The results presented above are approximately consistent with those of this meta-analysis, indicating that IBSA is not inferior to ABT and may even be better than ABT. In comparison, this review included 12 studies and provided the first comprehensive meta-analysis of effect of IBSA on clinical prognosis after LT for HCC, due to the lack of data, this analysis mainly focused on the clinical prognosis of IBSA. Predesigned subgroup analyses were conducted to evaluate whether the results were different with the use of LDFs. Multiple methods were adopted for sensitivity analyses, funnel plot and Egger regression test were used to estimate publication bias, which demonstrated the validity and robustness of the meta-analysis.

Several limitations of our study should be mentioned. First, the included studies were retrospective research and selection bias should not be ignored, since no RCT research on this question has been found after searching the databases. Well-designed, randomized, controlled, prospective trials are urgently required to clarify the existing concerns. Second, we only included English language studies due to the constraints of translating foreign language studies. Third, the included studies did not explore the use of allogeneic blood products, which may affect survival outcomes and prognosis due to their impact on immunity. Moreover, although significant heterogeneity was not found, patients’ characteristics varied across included studies. Only part of included studies use a propensity score to control for the effect of confounding and address selection bias, more detailed subgroup analyses were difficult to conduct, because of multiple outcomes and insufficient studies.

## Conclusion

These 12 studies represent the best reliable evidence to date. This meta-analysis may at least indicate that intraoperative blood salvage autotransfusion provided comparable survival outcomes relative to allogeneic blood transfusion and did not increase the tumor recurrence for hepatocellular carcinoma patients after liver transplantation. A reappraisal of the appropriate strategy for blood management during liver transplantation is warranted. High quality researches are required in the future to provide more sufficient evidence.

## Data availability statement

The original contributions presented in the study are included in the article/**Supplementary Material**. Further inquiries can be directed to the corresponding author.

## Author contributions

ZW developed the initial idea for this study. SL and KY developed and revised the search strategy. MS and ML finished the study design. KY and KL carried out data extraction and assessment of risk of bias. ZW, SL and YJ contributed to the original draft. DL and KL and were responsible for the revision of the draft. All of the authors approved the final work prior to submission ZW, SL and YJ have contributed equally to this work. All authors contributed to the article and approved the submitted version.

## Acknowledgments

The authors thank all the authors of the original studies included in this meta-analysis.

## Conflict of interest

The authors declare that the research was conducted in the absence of any commercial or financial relationships that could be construed as a potential conflict of interest.

## Publisher's note

All claims expressed in this article are solely those of the authors and do not necessarily represent those of their affiliated organizations, or those of the publisher, the editors and the reviewers. Any product that may be evaluated in this article, or claim that may be made by its manufacturer, is not guaranteed or endorsed by the publisher.

## Supplementary material

Supplementary Figure 1 Meta-analysis forest plot of the overall survival after sensitivity analysis. **(A)**, 1-year OS; **(B)**, 3-year OS; **(C)**, 5-year OS.

Supplementary Figure 2 Funnel plot of publication bias test for RR outcomes. Upper left, 1-year RR; Upper right, 3-year RR; Lower left, 5-year RR; Lower right, 7-year RR.

Supplementary Figure 3 Funnel plot of publication bias test for OS outcomes. Left, 1-year OS; Middle, 3-year OS; Right, 5-year OS.

## Abbreviations

ABT, allogenic blood transfusion; CI, confidence interval; DFS, disease-free survival rate; HCC, hepatocellular carcinoma; IBSA, intraoperative blood salvage autotransfusion; LDFs, leukocyte depletion filters; LT, liver transplantation; OR, odds ratio; OS, overall survival outcome; PROSPERO, International Prospective Register of Systematic Review; RCTs, randomized controlled trials; RR, the recurrence rate.
