# Supplementary material for: Clinical prognosis of intraoperative blood salvage autotransfusion in liver transplantation for hepatocellular carcinoma: A systematic review and meta-analysis
Source: Front Oncol. 2022 Oct 18;12:985281. doi: 10.3389/fonc.2022.985281 (PMC9622948; doi:10.3389/fonc.2022.985281)
Supplement: Supplementary file 1 [file DataSheet_1.docx]

| Search step | Search terms |
| --- | --- |
| #1 | "blood transfusion, autologous"[MeSH Terms] OR "autologous blood transfusion"[Title/Abstract] OR "transfusions autologous blood"[Title/Abstract] OR "blood transfusions autologous"[Title/Abstract] OR "transfusion autologous blood"[Title/Abstract] OR "Autotransfusion"[Title/Abstract] OR "Autotransfusions"[Title/Abstract] OR "autologous blood transfusions"[Title/Abstract] |
| #2 | "liver transplantation"[MeSH Terms] OR "grafting liver"[Title/Abstract] OR "liver grafting"[Title/Abstract] OR "transplantation liver"[Title/Abstract] OR "liver transplantations"[Title/Abstract] OR "liver transplant"[Title/Abstract] OR "liver transplants"[Title/Abstract] OR "transplant liver"[Title/Abstract] OR "hepatic transplantation"[Title/Abstract] OR "hepatic transplantations"[Title/Abstract] OR "transplantation hepatic"[Title/Abstract] |
| #3 | "carcinoma, hepatocellular"[MeSH Terms] OR "carcinomas hepatocellular"[Title/Abstract] OR "hepatocellular carcinomas"[Title/Abstract] OR (("carcinoma, hepatocellular"[MeSH Terms] OR ("Carcinoma"[All Fields] AND "Hepatocellular"[All Fields]) OR "hepatocellular carcinoma"[All Fields] OR ("Liver"[All Fields] AND "Cell"[All Fields] AND "Carcinoma"[All Fields]) OR "liver cell carcinoma"[All Fields]) AND "Adult"[Title/Abstract]) OR "liver cancer adult"[Title/Abstract] OR "adult liver cancer"[Title/Abstract] OR "adult liver cancers"[Title/Abstract] OR (("cancer s"[All Fields] OR "cancerated"[All Fields] OR "canceration"[All Fields] OR "cancerization"[All Fields] OR "cancerized"[All Fields] OR "cancerous"[All Fields] OR "neoplasms"[MeSH Terms] OR "neoplasms"[All Fields] OR "Cancer"[All Fields] OR "Cancers"[All Fields]) AND "adult liver"[Title/Abstract]) OR (("cancer s"[All Fields] OR "cancerated"[All Fields] OR "canceration"[All Fields] OR "cancerization"[All Fields] OR "cancerized"[All Fields] OR "cancerous"[All Fields] OR "neoplasms"[MeSH Terms] OR "neoplasms"[All Fields] OR "Cancer"[All Fields] OR "Cancers"[All Fields]) AND "adult liver"[Title/Abstract]) OR (("Liver"[MeSH Terms] OR "Liver"[All Fields] OR "livers"[All Fields] OR "liver s"[All Fields]) AND "cancers adult"[Title/Abstract]) OR "liver cell carcinoma"[Title/Abstract] OR "carcinoma liver cell"[Title/Abstract] OR (("Carcinoma"[MeSH Terms] OR "Carcinoma"[All Fields] OR "Carcinomas"[All Fields] OR "carcinoma s"[All Fields]) AND "liver cell"[Title/Abstract]) OR "cell carcinoma liver"[Title/Abstract] OR "cell carcinomas liver"[Title/Abstract] OR "liver cell carcinomas"[Title/Abstract] OR "hepatocellular carcinoma"[Title/Abstract] OR "Hepatoma"[Title/Abstract] OR "Hepatomas"[Title/Abstract] |
| #4 | #1 AND #2 AND #3 |
