# Supplementary material for: Clinical prognosis of intraoperative blood salvage autotransfusion in liver transplantation for hepatocellular carcinoma: A systematic review and meta-analysis
Source: Front Oncol. 2022 Oct 18;12:985281. doi: 10.3389/fonc.2022.985281 (PMC9622948; doi:10.3389/fonc.2022.985281)
Supplement: Supplementary file 3 [file Table_2.docx]

**Supplementary Table. The Newcastle-Ottawa Scale Quality Assessment of the eligible studies**

| **Study** | **Selection of cohorts** | | | | **COMPARABILITY** | **OUTCOME** | | | **NOS**  **Score** |
| --- | --- | --- | --- | --- | --- | --- | --- | --- | --- |
|  | Representativeness of the Exposed Cohort^1^ | Selection of the Non-Exposed Cohort | Ascertainment of Exposure^2^ | Demonstration That Outcome of Interest Was Not Present at Start of Study | Comparability of Cohorts on the Basis of the Design or Analysis^3^ | Assessment of Outcome | Was Follow-Up Long Enough for Outcomes to Occur^4^ | Adequacy of Follow Up of Cohorts^5^ |  |
| Akbulut et al（2013） | **☆** | **☆** | **☆** | **☆** | **☆** | **☆** | **☆** |  | 7 |
| Araujo et al （2016） | **☆** | **☆** | **☆** | **☆** | **☆☆** | **☆** | **☆** |  | 8 |
| Foltys et al（2011） | **☆** | **☆** | **☆** | **☆** | **☆☆** | **☆** | **☆** |  | 7 |
| Han et al（2016） | **☆** | **☆** | **☆** | **☆** | **☆☆** | **☆** | **☆** | **☆** | 9 |
| Ivanics et al （2021） | **☆** | **☆** | **☆** | **☆** | **☆☆** | **☆** | **☆** |  | 8 |
| Kim et al （2012） | **☆** | **☆** | **☆** | **☆** | **☆** | **☆** | **☆** | **☆** | 8 |
| Kwon et al（2021） | **☆** | **☆** | **☆** | **☆** | **☆** | **☆** | **☆** | **☆** | 8 |
| Muscari et al （2005） | **☆** | **☆** | **☆** | **☆** |  | **☆** | **☆** | **☆** | 7 |
| Nutu et al （2021） | **☆** | **☆** | **☆** | **☆** | **☆☆** | **☆** | **☆** |  | 8 |
| Pinto et al （2021） | **☆** | **☆** | **☆** | **☆** | **☆** | **☆** | **☆** |  | 7 |
| Sutton et al （2021） | **☆** | **☆** | **☆** | **☆** | **☆** | **☆** | **☆** |  | 7 |
| Weller et al （2021） | **☆** | **☆** | **☆** | **☆** |  | **☆** | **☆** |  | 6 |

Total score was 9 stars for each study. A study can be awarded a maximum of one star for each numbered item within the Selection and Exposure categories. A maximum of two stars can be given for Comparability.

1 Exposure was referred to patients with hepatocellular carcinoma who underwent liver transplantation for the first time.

2 One star would be given for secure record (eg surgical records).

3 If a study was adjusted for blood loss during LT, one star was awarded, and if a study adjusted for any additional factors (e.g. preoperative hemoglobin, preoperative platelet count, preoperative international normalized ratio, Child– Pugh score, allograft type, warm ischemia time, previous abdominal surgery, recipient age, etc.), an additional star was awarded.

4 One star would be given for studies demonstrating the results of RR OS or DFS.

5 One star would be provided for studies with a follow-up rate of exceeding 50%. No statement would give zero star.

NOS, Newcastle-Ottawa Scale.
