# Supplementary figures and images for: Clinical prognosis of intraoperative blood salvage autotransfusion in liver transplantation for hepatocellular carcinoma: A systematic review and meta-analysis
Source: Front Oncol. 2022 Oct 18;12:985281. doi: 10.3389/fonc.2022.985281 (PMC9622948; doi:10.3389/fonc.2022.985281)

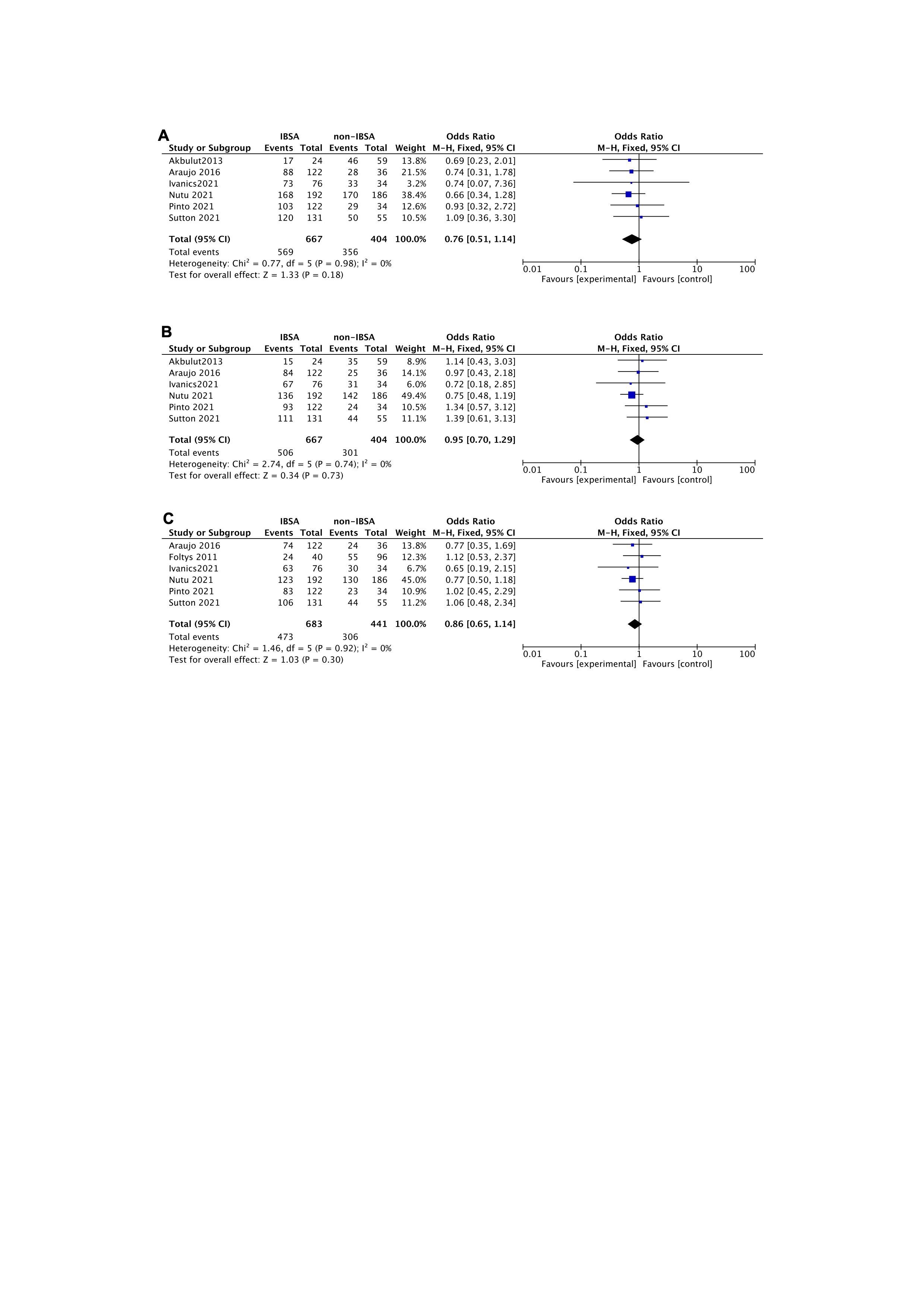

Supplement: Supplementary Figure 1 — Meta-analysis forest plot of the overall survival after sensitivity analysis. (A), 1-year OS; (B), 3-year OS; (C), 5-year OS. [file Image_1.jpeg]

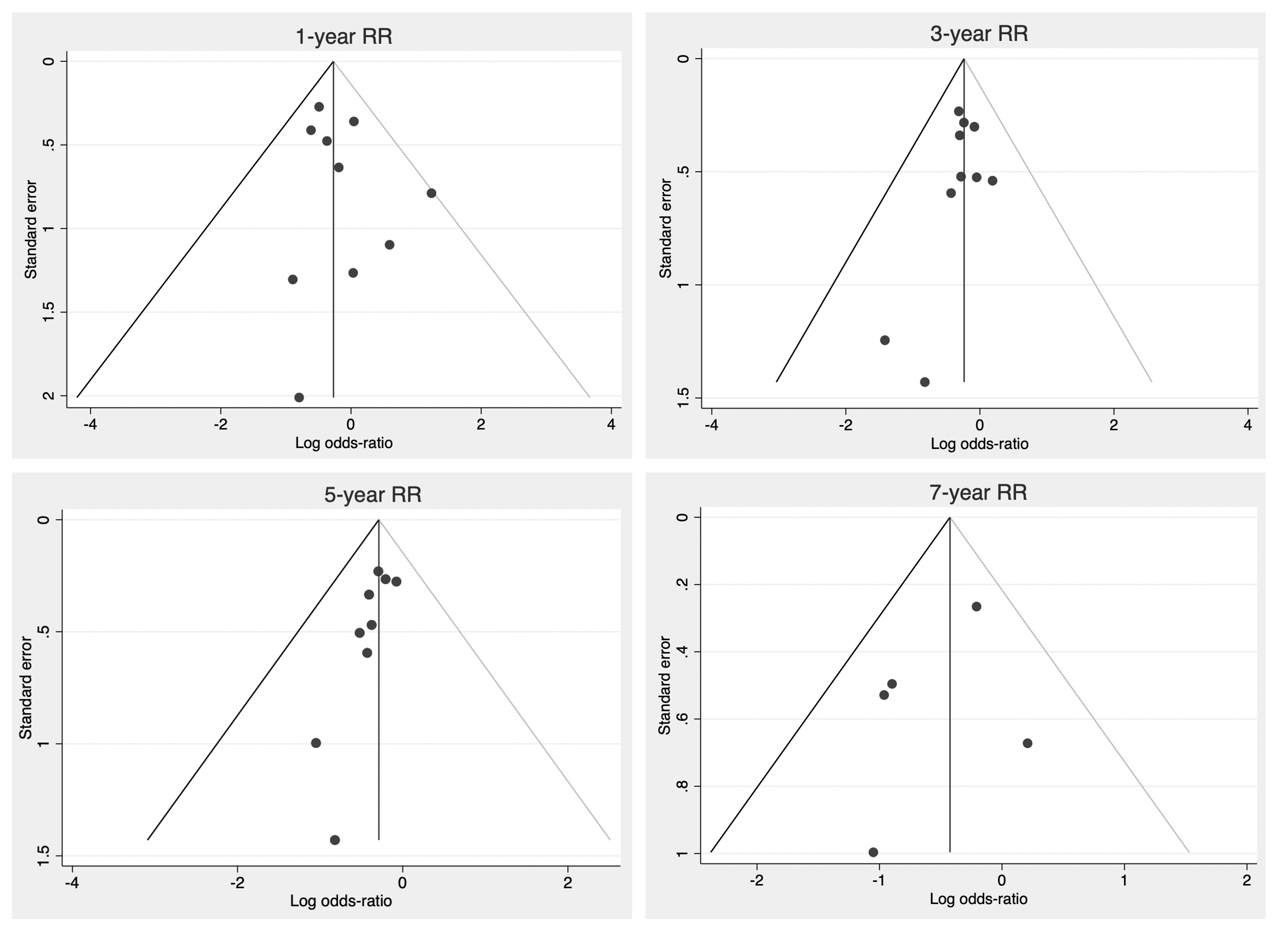

Supplement: Supplementary Figure 2 — Funnel plot of publication bias test for RR outcomes. Upper left, 1-year RR; Upper right, 3-year RR; Lower left, 5-year RR; Lower right, 7-year RR. [file Image_2.jpeg]

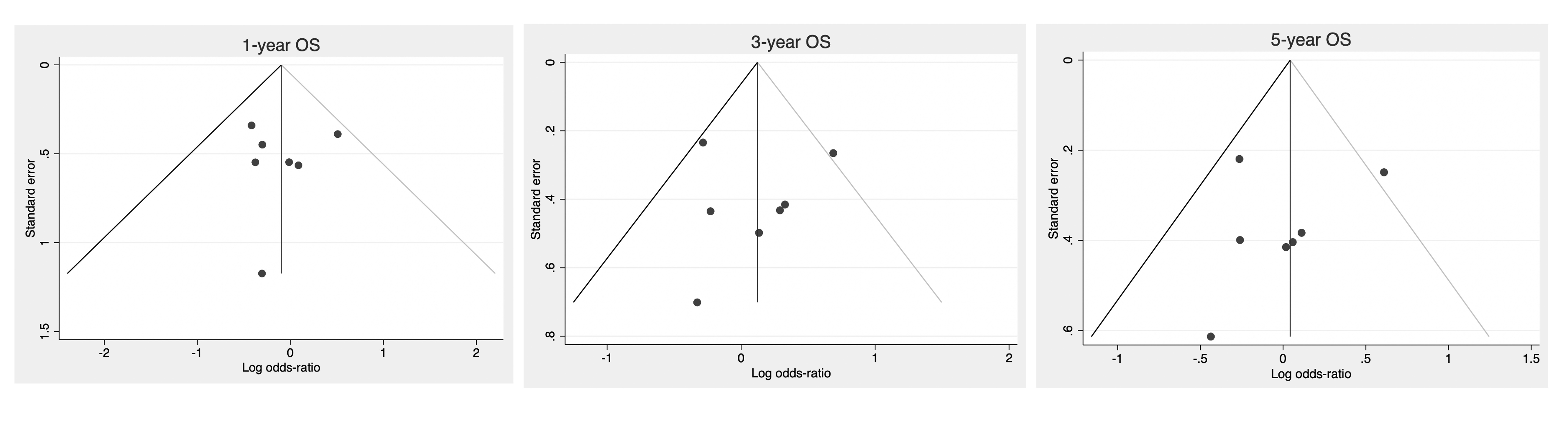

Supplement: Supplementary Figure 3 — Funnel plot of publication bias test for OS outcomes. Left, 1-year OS; Middle, 3-year OS; Right, 5-year OS. [file Image_3.jpeg]
